# A Case Report of Successful Medical Management of Emphysematous Gastritis With Portal Venous Air

**DOI:** 10.7759/cureus.25095

**Published:** 2022-05-18

**Authors:** Anass Dweik, Waqas Rasheed, Basheer U Mohammed, Michael W Ebrahim, Omar Bazzaz

**Affiliations:** 1 Internal Medicine, Texas Tech University Health Sciences Center, Amarillo, USA; 2 Internal Medicine, Thomas E. Creek Veterans Affairs Medical Center, Amarillo, USA

**Keywords:** case report, gastritis, gastric emphysema, portal venous air, medical management, emphysematous gastritis

## Abstract

Emphysematous gastritis is an uncommon yet life-threatening condition characterized by air in the gastric wall. It requires clinical and radiological findings for its diagnosis. Management is done either medically or surgically and requires early intervention with bowel rest, proton pump inhibitors, and antibiotics with or without gastrectomy secondary to the high mortality associated with the condition. We present a case of a 55-year-old male who responded well to medical therapy without the need for surgical intervention.

## Introduction

Emphysematous gastritis (EG) is an uncommon life-threatening condition that is characterized by air in the gastric wall caused by gas-forming microorganisms leading to systemic toxicity. It has a high mortality rate approaching 55-61% [1]. EG is associated with a plethora of conditions including chronic alcohol consumption, recent abdominal surgery, hematological malignancies, diabetes, gastric wall corrosion from alkali or acid poisoning, phytobezoar consumption, and long-term treatment with steroids or antibiotics [2]. There are no specific guidelines with regard to the treatment or management approach; nevertheless, early diagnosis along with hydration, intravenous (IV) antibiotics, proton pump inhibitors (PPIs), and bowel rest have shown to improve survival [3]. We present a case of a 55-year-old male admitted with EG who responded completely to medical management with IV fluids, antibiotics, and PPI.

## Case presentation

**Figure 1:**
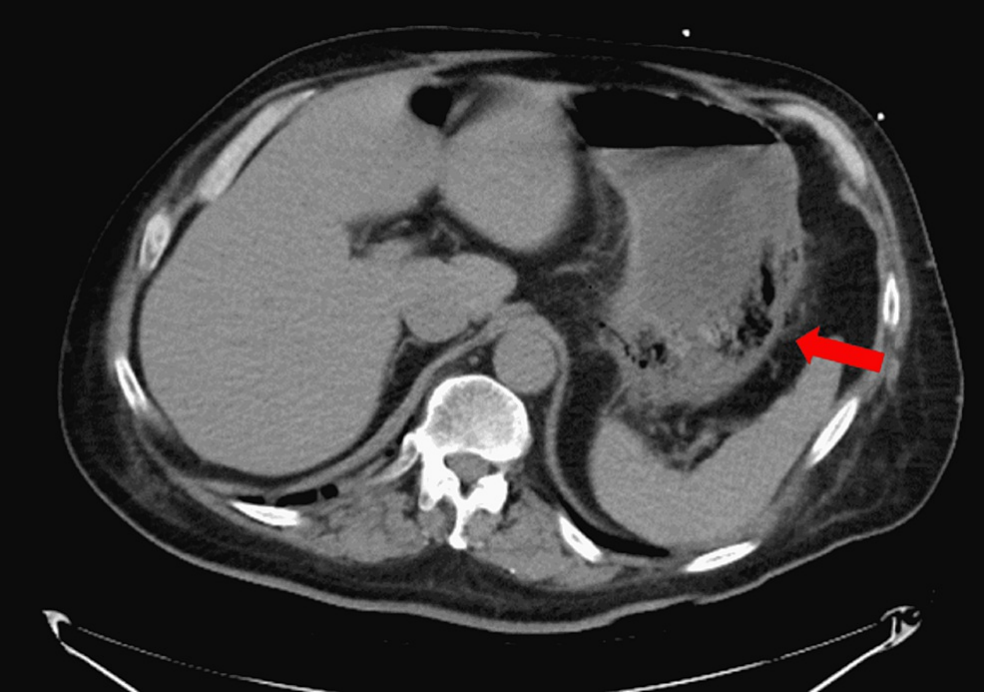
Evidence of pneumatosis involving the proximal gastric wall that was seen on admission

**Figure 2:**
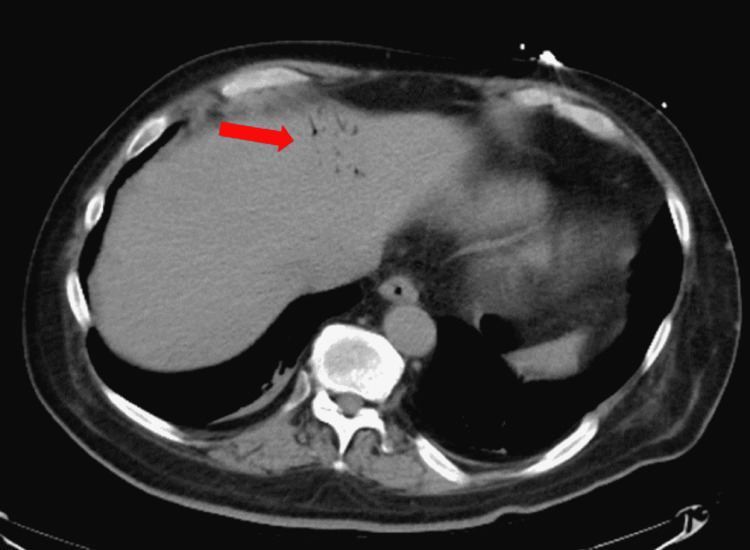
Portal venous air seen on admission

**Figure 3:**
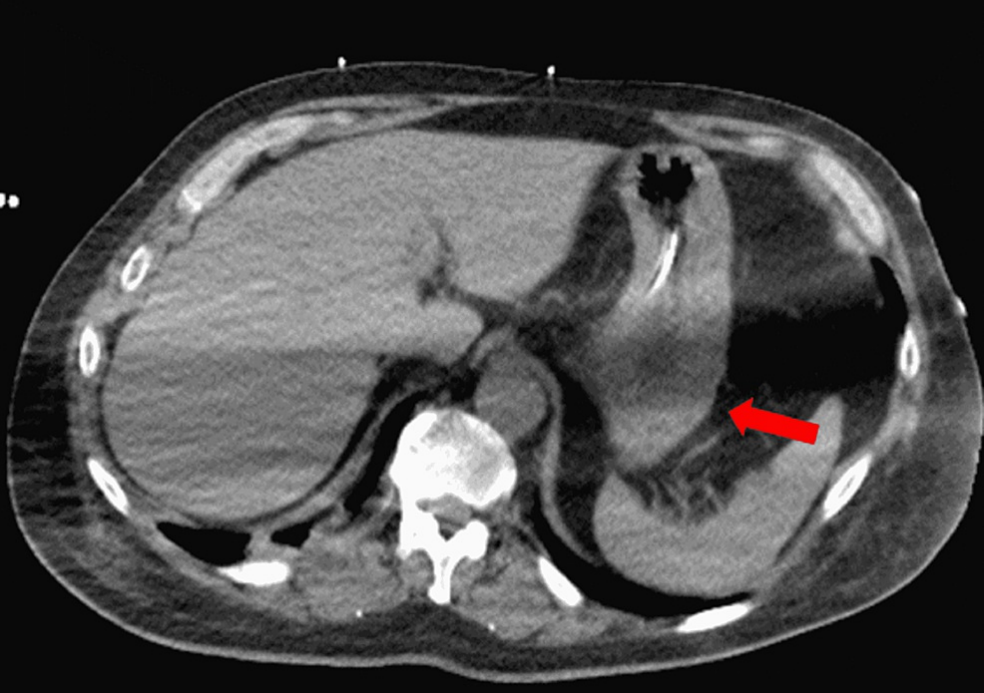
The resolution of the previously seen proximal gastric wall pneumatosis on repeat imaging

**Figure 4:**
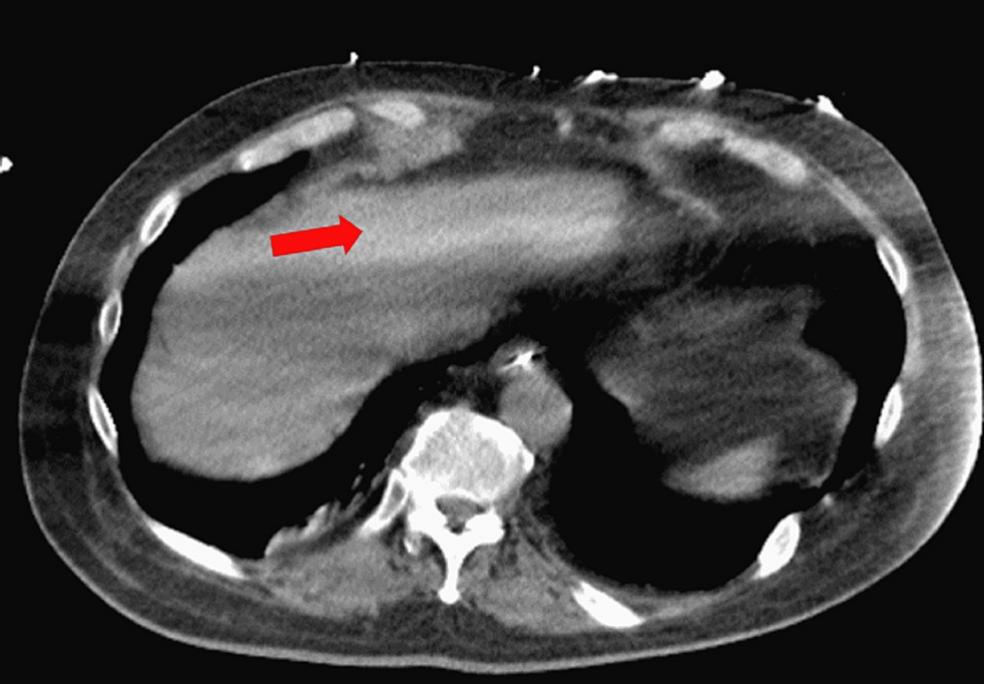
The resolution of the previously seen portal venous air on repeat imaging

A 55-year-old male with an extensive medical history including hypertension, diabetes, adrenal insufficiency, quadriplegia, and dysautonomia secondary to C7 fracture from a motor vehicle collision (MVC) 12 years prior presented with nausea and coffee-ground emesis along with abdominal distention and bloating. Symptoms started a day prior to his admission. He denied tobacco, alcohol, or illicit drug use. Initial vital signs revealed blood pressure of 116/91 mmHg, heart rate of 109 beats/minute, respiratory rate of 20 breaths/minute, and a body temperature of 37.9°C. Physical examination was significant for abdominal distention with hyper-resonance at the left upper quadrant with appropriate bowel sounds. He had a left lower quadrant colostomy that looked clean and had no signs of active infection or inflammation. His colostomy was done following his MVC as a precautionary measure to prevent infections from decubitus ulcers, as he was bed-bound. Laboratory studies were significant for a hemoglobin level of 14 g/dl, with normal prothrombin time, partial thromboplastin time, and international normalized ratio. A stool occult blood test was positive. An abdominal CT scan was done on admission and showed gastric distention with evidence of pneumatosis involving the proximal gastric wall along with some degree of fluid and air, suggesting EG (Figure 1). In addition, the liver demonstrated portal venous air (Figure 2). Surgery and gastroenterology were consulted and recommended to place a nasogastric tube (NGT) on intermittent suction along with IV pantoprazole and piperacillin-tazobactam 4.5 g every eight hours. His hemoglobin dropped to 12 g/dl on the second day of hospitalization but remained stable thereafter. The patient’s symptoms improved over time with the resolution of bloating, abdominal distention, and hematemesis. On his fourth day of admission, CT of the abdomen was repeated and it showed resolution of the previously seen EG and portal venous air (Figures 3, 4). Antibiotics were continued for a total of seven days and were later discontinued. Pantoprazole was also switched from IV to oral tablets. The patient’s hospitalization was prolonged due to worsening dysautonomia; however, his gastrointestinal symptoms did not recur.

## Discussion

Gastric emphysema can be generally categorized based on its etiology including gastric and extragastric causes. Gastric causes include conditions such as EG that is related to infections, ischemia, caustic ingestion, increased intraluminal pressure such as in the case of severe vomiting or bowel obstruction, and perforated gastric ulcer. Extragastric causes include bowel ischemia from either small or large intestine, gangrenous cholecystitis, or very rarely from dissection of pulmonary bulla [4]. One of the most important rule-outs in the workup of gastric emphysema is EG due to its high mortality rate. EG is caused by bacterial infections that may arise either locally through the mucosa or through hematogenous seeding from a distal nidus [5]. Gastric mucosal lining is known to have a rich blood supply and a low pH, both of which help in forming a resistant barrier to infections underlining the importance of a trigger for infections to interrupt the integrity [6]. A detailed history is always needed to help identify the trigger. Some of the most common triggers include chronic alcohol consumption, recent abdominal surgery, hematological malignancies, diabetes, gastric wall corrosion from alkali or acid poisoning, phytobezoar consumption, and long-term treatment with steroids or antibiotics [2]. Our patient had three triggers, which were a history of diabetes and long-term treatment with steroids and antibiotics. That was mainly because he had adrenal insufficiency that required steroids and he also had a chronic indwelling suprapubic catheter that was placed following the development of quadriplegia. Moreover, he had severe vomiting on admission; however, it was not clear if his vomiting preceded the development of EG.

Patients can present with any of the following symptoms including abdominal pain, nausea with or without vomiting, hematemesis, and fever. Typical bedside examination findings include abdominal distention and diminished bowel sounds, which our patient also presented [1]. Diagnosis is made with an abdominal CT that would show irregular gas patterns in the gastric wall, which may spread to perigastric vessels including the portal vein [6,7]. Our patient did have both findings present on his initial CT scan. Medical management has been the trend since the early 2000s and the mortality rate has been declining with the overall advances in health care. Nevertheless, surgery is indicated in patients who continue to deteriorate despite optimal medical management [6]. Thankfully, our patient tolerated medical management well with hydration, IV antibiotics, and bowel rest. Moreover, we were able to prove the resolution of his condition with a repeat CT scan during his hospitalization.

## Conclusions

In conclusion, EG is a serious condition that can present with a wide range of symptoms and is associated with multiple conditions. It is important to identify the condition at its early stages to help lower the mortality risk. Overall, previous trends used to favor surgical intervention in efforts of treating the condition given its high mortality rate. However, multiple case reports over the last century were able to prove the success of medical management. Our case presented herewith was treated successfully with medical therapy without the need for surgical intervention. We hope that international societies do shed light on this uncommon yet serious condition with formal guidelines to help lower the reported mortality rate.
